# Strategies to Approach Medicines Litigation: An Action Research Study in Brazil

**DOI:** 10.3389/fphar.2021.612426

**Published:** 2021-04-22

**Authors:** Fernanda Lacerda da Silva Machado, Danielle Maria de Souza Serio dos Santos, Luciane Cruz Lopes

**Affiliations:** ^1^ Federal University of Rio de Janeiro, UFRJ, Macaé, Brazil; ^2^ Pharmaceutical Sciences Graduate Course, University of Sorocaba, UNISO, Sorocaba, Brazil

**Keywords:** pharmaceutical services, health’s judicialization, right to health, action research, essential medicines, educational outreach visits, group conversations

## Abstract

**Background:** In the last decades, litigation has been increasingly used to access medicines in Brazil. This phenomenon has led to the development of diverse strategies to reduce its negative impact on the organization of pharmaceutical services. In spite of that, managers still face difficulties dealing with lawsuits.

**Objective:** This study aims to report the planning and implementation of strategies to approach medicines litigation in a municipality located in the southeast region of Brazil.

**Methods:** Mixed methods were employed through an action research cycle. A network coordination team included researchers from university and municipal managers. The scenario analysis comprised the characterization of pharmaceutical services and the profile of medicines lawsuits. Afterward, strategies were planned to deal with the central problem identified. The action plan involved educational outreach visits and distribution of printed materials for health professionals, evaluated through opinion survey. Group conversations were conducted with the users of the public health system, followed by thematic analysis of reports.

**Results:** The characterization of pharmaceutical services in the municipality revealed that treatments supplied were in accordance with the National Medicines Policy. In addition, a sector was implemented to attend demands for non-incorporated medicines. In spite of the services available, the characterization of lawsuits indicated that the main claimants were users of the public health system, requiring non-incorporated medicines, with therapeutic alternatives available. Thus, educational outreach visits were held in 14 health units (23 physicians in total). Everyone who answered the evaluation declared that they were very satisfied with the approach. Group conversations with the users of the health system reached 227 participants in total. In regard to users’ perception about pharmaceutical services, thematic analysis of reports identified three main categories including aspects related to medicines provided, users assisted, and quality of service.

**Conclusion:** The study described the first cycle of an action research project to develop strategies to approach medicines litigation at the municipal level. The application of educational outreach visits for health professionals and group conversations with health system users is a promising approach to improve access to information about pharmaceutical services in Brazil.

## Introduction

The lawsuits for access to medicines impact the structure, financing, and organization of healthcare services as they neglect the priorities established through public policies and force reallocation of financial and human resources with the potential to widen inequality (Wang, 2015). Brazil has faced a marked increase in the expenditure on claims ordered by litigation since 2005 (Cubillos et al., 2012). Data from the Ministry of Health estimated that from 2009 to 2016, the amount allocated to deal with lawsuits rose approximately 13 times, reaching more than US$ 293 million (Ayres et al., 2018).

Therefore, efforts have been made to mitigate the negative impacts on the public health system by different institutions involved with the phenomenon. In response to litigation, health system managers had to remodel and structure new services to address the increasing demand of lawsuits to access medicines (Catanheide et al., 2016).

In Brazil, the institutional response of the judicial branch included the Recommendation 31/2010 (Conselho Nacional de Justiça, 2010) issued by the National Council of Justice, which advises courts to implement actions to support judges. These included the requirement of detailed medical reports to support decision-making, encouraging judicial workers to visit health units, and the establishment of interinstitutional agreements to provide health workers support in assessing the clinical issues presented by the claimants. In conjunction, the aim of these measures was to raise the courts’ awareness about healthcare policies giving a broader perspective of the system in order to enable better decisions.

From the legislative branch, the Federal Law 12.401/2011 (Brasil, 2011a) clarifies the definition of the right to health in the Constitution stating that integral access to medicines in the public health system encompasses the provision of treatments according to the funded lists and the clinical protocols and therapeutic guidelines issued by the Ministry of Health. However, previous studies suggest that part of the lawsuits could be prevented if the regulations of the public health system were adhered to (Carvalho and Leite, 2014; Lopes et al., 2014; Catanheide et al., 2016; Freitas et al., 2020).

Despite the current pharmaceutical policy and the strategies implemented by the legislation, medicines litigation is still a challenging topic for the various actors involved (Carvalho and Leite, 2014; Biehl et al., 2019; Vargas-Pelaez et al., 2019). It is believed that through action research in which actors are involved cooperatively, it could contribute to the planning and implementation of strategies in the local context for a better approach to medicines litigation in a municipality located in southeastern Brazil.

## Methods

### Design and Setting

This study used mixed methods with an action research design. This approach combines action and reflection, and it is recommended to identify deficiencies in the health services with a key aspect of allowing the emancipation of subjects, through their involvement in research and shared construction of knowledge (Thiollent, 2011).

The study site was a municipality in the southeastern region of Brazil, located in the state of Rio de Janeiro with a population of 230,000 inhabitants (IBGE, 2016). The health secretary and the pharmaceutical services (PS) department of the municipality, as well as many others in Brazil, faced a large number of litigation cases in health, especially related to medicines. To deal with this reality, these sectors contacted a local university to establish a university–community partnership to come up with strategies to improve this scenario.

### The Network Coordination Team and the Research Plan

The network coordination team was composed of seven pharmacists: three researchers from the university and four municipal managers. The former with experience in public health and direct involvement in outreach projects, while the city managers have had hands-on experience working for at least 5 years in the PS department.

The network coordination team initiated the diagnosis (scenario analysis) by identifying the central problem, followed by a planning phase of the strategy to be implemented. Finally, the action research cycle involved the application of the developed strategy, its evaluation by health professionals, and reflection by the team.

### Scenario Analysis

The identification of the central problem related to medicines litigation involved two main actions: an analysis of the organization of PS and the profile of litigated medicines in the municipality. To understand how PS was structured, the research group analyzed the municipality’s electronic address to check the legal documents available and current actions developed by the PS sector. The identified actions were classified according to the categories presented by Yamauti et al. (2020).

To characterize the profile of medicines litigation, data were collected in the public pharmacy responsible for dispensing non-incorporated medicines to users of the municipal health system. All information collected were in accordance with the Brazilian Law of Access to Information (Law 12.527/2011) (Brasil, 2011b), which guarantees privacy of user identification. For this data collection, the eligibility criteria were: inclusion of all lawsuits against the public health system that were in force in March 2018 requiring at least one item registered as a medicine. In all lawsuits described, claimants were fully attended with all medicines litigated, even if they were not regularly funded in the public health system.

The following variables were collected: lawsuit register number, date of request, age of the patient, gender, prescriber(s) registration number, medical specialty(s), the type of service that provided the prescription, legal representation of the plaintiff, lawyer’s registration number, presence of prescription and medical report in the lawsuit, medicine(s), dosage form, and clinical indication. Descriptive analysis of the data was performed by determining the absolute and relative frequencies (qualitative variables), means, medians, and standard deviation (quantitative variables).

The requested medicines were classified according to the Anatomical Therapeutic Chemical (ATC) classification system and the presence in one of the official lists of medicines funded by the municipality’s health system, namely, Brazilian National List of Essential Medicines (RENAME, in Portuguese), 2017 edition (Ministério da Saúde, 2017); medicines offered by the State Secretary of Health (Secretaria Estadual de Saúde do Rio de Janeiro, 2018); and the Municipal List of Essential Medicines (REMUME, in Portuguese) (Prefeitura Municipal de Macaé, 2017). Additionally, medicines not listed in Brazilian lists were compared to the Model List of Essential Medicines (MLEM) from the World Health Organization (World Health Organization, 2017).

In order to better understand the reasons for the prescription of non-incorporated medicines, therapeutic alternatives were evaluated according to their availability in at least one of the lists. This analysis was according to the Manual of Indicators of Evaluation and Monitoring of Medicines Lawsuits, which suggests considering medicines in the same pharmacological subgroup at the ATC system as an alternative (Pepe et al., 2011).

### Planning Phase and Application of the Strategy

**FIGURE 1:**
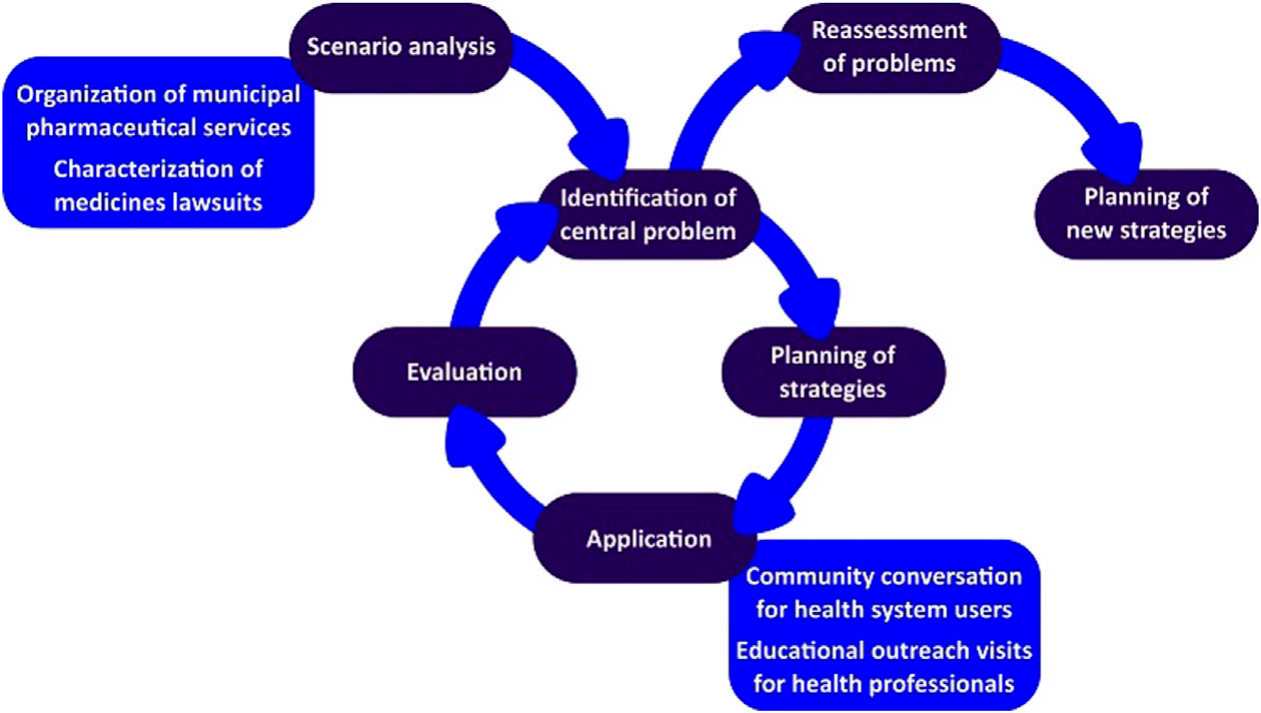
Action research cycle.

The planning phase was guided by the central problem identification related to medicines litigation, and possible gaps in the PS organization were listed for which hypotheses were proposed to help the development of an action plan. The defined action plan involved educational interventions, both for health professionals and users of the municipal public health system (Figure 1).

For health professionals, planned educational visits based on the principles of educational outreach, also known as academic detailing, and the development of printed educational materials were adopted. Academic detailing consists of educational encounters led by a trained facilitator held at the health unit aiming to stimulate behavioral changes, including the prescriptions practices (Kunstler et al., 2019). Undergraduate pharmacy students were recruited to carry out the visits, having received training promoted by the network coordination team. It should also be noted that students were always accompanied by graduated professionals. All health professionals who provided medical care at specialized centers responsible for medicine prescription to users identified as relevant to the central problem in question and who agreed to participate in the educational visit were considered eligible for this. Thus, for this activity, the sample was for convenience, from contact with 19 specialized health centers.

For the health system users, group conversations were held at Family Health Strategies (ESF, in Portuguese), which are Brazilian health units responsible for primary health care. The group conversations aimed to discuss PS organization in the SUS, especially how to access medicines in the public health system of the municipality. For the activity, a semi-structured script was proposed (Supplementary Appendix 1), and two students previously trained for this activity conducted it. One was the mediator, who facilitated the discussions, encouraging the free expression of ideas, and the other one the observer, who registered doubts and opinions presented. Based on these notes, a report of the group conversation was prepared using a standardized document with fields to include date, location, team that was present in the activity, moderator, observer, number of participants, general characteristics (gender and age group), duration, and one field for detailed description of the activity. The sample of this activity was also for convenience, and all ESF in the city, 44 in total, were invited, in those which contact and acceptance were possible, the activity was carried out.

### Evaluation

For the educational outreach visits, at the end of the visits, the professionals were invited to fill out an evaluation form, which included six questions addressing 1) clarity of the proposal’s presentation, 2) duration of the visit, 3) appropriateness of the material, 4) clarification of doubts, 5) posture of the student, and 6) verbal expression. A 5-point Likert scale was used, ranging from very dissatisfied to very satisfied. The evaluation form also included three open questions that allowed the health professional to report suggestions or additional comments (Supplementary Appendix 1).

In regard to group conversations, each report produced by the observer was reviewed and, if necessary, complemented by the other team participants who were present at the conversation. Thematic analysis of these reports was led with an inductive approach (Braun and Clarke, 2006). The first author, who had participated in all group conversations, conducted coding and identification of themes. Then, the results were discussed and reviewed by the second author.

### Ethical Aspects

This study is approved by the Research Ethics Committee of the Federal University of Rio de Janeiro—Campus Macaé (Protocol number: 4.187.153)—and respected the ethical protocols recommended in Resolution no 466/2012 (Brasil, 2012) of the National Health Council, which includes the Regulatory Guidelines for Research involving human beings.

## Results

### Organization of Pharmaceutical Services

In Brazil, medicines funded by SUS are divided into three groups, called components, namely, basic, specialized, and strategic. The first encompasses medicines employed at primary health care, mainly acquired by the municipal government; the second includes high-cost medication, managed by each state government; and the strategic component, which comprises treatments for diseases with potential endemic impact and under federal responsibility (Ministério da Saúde, 2019).

According to the National Medicines Policy, RENAME is the main reference for medicines selection in the public system. Municipalities are responsible for electing medicines according to the epidemiology of diseases affecting their citizens, resulting in the REMUME. At the time of the research, the REMUME had been updated in 2017, including 211 medicines for primary health care (Prefeitura Municipal de Macaé, 2017), 80.6% of it corresponded to products included in the national list. In addition, 77.2% of REMUME were listed on MLEM (World Health Organization, 2017).

PS also provided access to medicines from the strategic and specialized components. For the latter, the municipality had one public pharmacy dispensing high-cost treatments, managed by the state government (Secretaria Estadual de Saúde do Rio de Janeiro, 2019). Therefore, the structure of PS was organized to provide treatments consistent with the regulations of the public health system.

**FIGURE 2:**
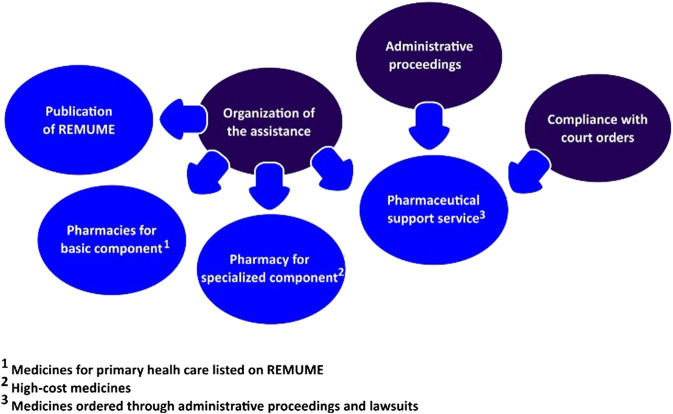
Strategies implemented by the Municipal Secretary of Health to deal with medicines litigation.

Not only did the municipal PS provide assistance for users claiming listed medicines but a special sector was also implemented to evaluate demands for non-incorporated products, called pharmaceutical support service. The sector was responsible for registering and evaluating requirements from administrative proceedings, in addition to attending cases of medicines lawsuits (compliance with court orders) (Prefeitura Municipal de Macaé, 2016). In regard to strategies to approach medicines litigation, it was observed that the PS department had implemented actions related to the organization of assistance, administrative proceedings, and compliance with court orders (Yamauti et al., 2020), as described in Figure 2.

### Problems Related to Medicines Litigation

In spite of the local organization of PS available at the public health system, lawsuits continue to be employed as a strategy to access pharmacological treatments in the municipality. The study identified and analyzed 82 lawsuits requiring medicines, which were litigated from 2005 to 2018. The full description of demands can be found in Supplementary Appendix 1.

In relation to the legal representation, 81.8% (*n* = 63) of the lawsuits were from the public sector, represented by the municipal public defender’s office and 18.2% were from private lawyers. Since the public defender’s office represents citizens of low income or high social vulnerability, the main litigants in the present study were from the lower social class.

In addition, litigants were mainly assisted by the public health system. In regard to the type of the service, 82.9% (*n* = 68) of lawsuits included prescription from public health units, 11.0% (*n* = 9) from private health units, and 6.1% (*n* = 5) from both.

The endocrine, nutritional, and metabolic diseases (ICD-10: Chapter IV) were the most frequent conditions requesting treatments, with higher frequency for diabetes mellitus (21, 25.6%), followed by the disorders of other endocrine glands (4, 4.9%). The second and third most frequent conditions were the mental and behavioral disorders (10, 12.2%) and the diseases of the nervous system (8, 9.8%), respectively.

The characterization of medicines required in the lawsuits according to the ATC classification system can be found in Supplementary Appendix 1. The lawsuits demanded 227 medicines, ranging from one to 15 medicines/patient (mean 2.77, SD = 2.57 medicines/patient), corresponding to 132 substances and 150 dosage forms. Insulins and analogues represented 26.0% of the pharmaceutical products litigated. By the time of this investigation, these medicines were not incorporated in SUS. The first evaluation conducted by the Ministry of Health concluded that there was no robust evidence that they were more effective than human insulin for the control of diabetes (CONITEC, 2017). However, long and fast-acting insulins were included in the latest version of RENAME and will be supplied for the treatment of diabetes mellitus type 1 (Ministério da Saúde, 2019).

Medicines litigation demanded mainly products outside lists of funding in SUS. Only seven lawsuits (8.5%) demanded just incorporated medicines, prescribed in accordance with the clinical criteria established by the Clinical Protocols and Therapeutic Guidelines (national documents that regulate the access of high-cost medicines). Among all medicines claimed, 60.7% were not incorporated in RENAME. In regard to presence in the local lists, only 13.3% of medicines were supplied by the State Secretary of Health and 20% were listed on the REMUME.

The majority of lawsuits disregarded the existence of therapeutic alternatives in SUS. Among the 91 non-incorporated medicines, it was possible to identify therapeutic alternatives for 72 of them (85.7%), and only four (4.4%) were considered essential according to the WHO MLEM. For diabetes mellitus, metformin, glibenclamide, and gliclazide were listed at REMUME as blood glucose–lowering drugs (ATC A10A), while human insulin and NPH (ATC A10A) were the insulins available in SUS. In regard to nervous system agents, five lawsuits demanded three antipsychotics (ATC N05A: aripiprazole, levomepromazine, and paliperidone) with nine therapeutic alternatives (chlorpromazine chloride, haloperidol, haloperidol decanoate, lithium carbonate, clozapine, olanzapine, quetiapine, risperidone, and ziprasidone). In addition, for the four antiepileptics (ATC N03A) litigated, 11 medicines were listed in RENAME (Supplementary Appendix 1). Nevertheless, based on the information available in most of the lawsuits, it was not possible to recognize if the listed medicines had been used previously by the litigant.

### Defining the Central Problem

In this context, medicines litigation predominantly demanded medicines unavailable in the public health system. Therefore, the network coordination team elected as the central problem for the first cycle of this action research the prescription of non-incorporated medicines with therapeutic alternatives in the public health system.

Since medicines litigation is a complex phenomenon, secondary influencing factors were also listed and will be addressed in future stages of action research: 1) the appropriateness of medicines offered in SUS and 2) quality of PS offered at the municipality. These issues were not initially considered a priority for the following reasons: listed medicines were in accordance with the National Medicines Policy, and the results do not suggest that the majority of litigation had been a result of problems related to the management of PS, which could be observed if lawsuits claimed mainly incorporated products.

### Planning and Application

Based on the central problem, the network coordination team proposed two hypotheses: 1) knowledge gap about medicines incorporated in SUS and 2) distrust in the available treatments. Since communication is a key issue on both hypotheses, strategies to improve access to information about the organization of PS, especially about incorporated medicines, were considered a priority. In order to strengthen primary health care, the network coordination team agreed to focus on wide dissemination of REMUME.

Educational visits to discuss about REMUME were led between August 2018 and December 2019 in 14 health units where 24 medical specialties offered care for SUS. In total, 23 physicians participated in the visits, in which 80 brochures and 70 posters were distributed for consultation of REMUME. Among the 23 visited health professionals, 11 filled in the evaluation form. Everyone declared that they were very satisfied with the printed materials, two suggested that the same approach should be employed to the medicines of the specialized component of PS, and two mentioned the relevance of the initiative for the population.

**FIGURE 3:**
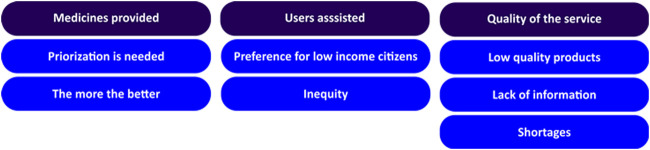
Categories identified through thematic analysis of reports of group conversations.

Group conversations with users of the public health system were conducted in 21 ESF. The discussions lasted from 20 to 60 min, varying from five to 23 participants, including 227 in total. In regard to participants’ perception about PS in the public health system, thematic analysis of reports identified three main categories, including aspects related to medicines provided, users assisted, and quality of service (Figure 3).

It was observed that participants of group conversations had different perspectives about medicines supplied in the public health system. Some argued that all medicines should be available, while others recognized the need of a prioritization process.

“The SUS, yes, it should give the treatment, the medicines that people need, because there are many medicines that we don’t have the money to buy.”

“I don’t think that (the government) would have a budget for everything, so they would have to focus on the specific ones.”

Demand on the prescriptions and cost (higher priority for the most expensive) were two criteria mentioned independently by participants during discussions to determine the importance of a specific medicine.

“I think the government should give the most expensive medicines, because we can buy a dipyrone.”

“It (the government) must supply the most used medicines that are prescribed by physicians”

Under the category of users assisted, participants claimed that low-income citizens should be preferably supported.

“They should analyze the person’s financial situation to provide medicine.”

“We must think of people who are unable to buy the medicines they need.”

In spite of that, equity was an issue raised during discussions. It was mentioned that non-incorporated medicines were prescribed only to people who could buy it because even if they were accessible by administrative proceedings or lawsuits, there is a risk of shortages in the public health system.

“Special medicines are only taken by people who are rich. For poor people, physicians only prescribe the cheap insulin”

In regard to quality of the service, a divergence in terms of satisfaction with PS in the municipality was also observed. While some declared that shortages were frequent, others affirmed that they had integral access to their treatments.

“Whenever I go to the pharmacy, I can never get my medicines.”

“When (medicine) is lacking in one day, the supply is soonly restarted.”

The lack of information also emerged during discussions, either of the specialized component and the “Programa Farmácia Popular” (in Portuguese). The latter was created by the Brazilian government to complement access to medicines through provision of free or cofinancing items in private pharmacies affiliated to the program.

“It was good, the Farmácia Popular I had already seen, but I didn't know I could get medicines for free.”

“My husband uses atorvastatin and I buy it every month, I didn’t know it was available on the public system.”

In addition, participants were frequently unsure about the quality of medicines provided. Some of them believe that products supplied in the public health system have lower quality and even reported that some physicians recommended them to buy, instead of taking it in the public pharmacy. Similarly, generic medicines were also perceived as less effective than brand products.

“Some physicians ask us to buy, because the medicines given by SUS do not work.”

“It is not possible to have this huge difference in price and have the same effect (generic medicines).”

## Discussion

The misconceptions about the right to health definition in the Brazilian Constitution result in judicial decisions that privilege individual claims, obligating managers to provide all medicines required (Wang, 2015). As a consequence, lawsuits can generate inequities since the distribution of resources will be related to the user’s litigation capacity. Thus, the present study described the first cycle of an action research project to develop strategies to approach medicines litigation at the municipal level.

Essential medicines lists are worldwide used as a key strategy to enable universal health coverage; however, funded products may vary significantly between countries (Persaud et al., 2019). In Brazil, the incorporation of medicines in the public system has evolved with the creation of CONITEC (National Council for Incorporation of Technologies in the National Health System) in 2011. Since then, the number of medicines provided in the public health system has been increasing (Yamauti et al., 2017), yet this was not associated with a reduction in litigation (Chieffi et al., 2017; Oliveira et al., 2020).

It is claimed that despite the adequacy of the National Medicines Policy, the perception that needs to access medicines in Brazil are not being fulfilled leads to the increasing use of lawsuits to provide treatments (Catanheide et al., 2016). Nevertheless, it could be argued that there is a gap between legislation and services.

Federal Law 12.401/2011 (Brasil, 2011a) established that the provision of medicines in SUS must follow the official medicines lists and Clinical Protocols and Therapeutic Guidelines. Thus, the litigation of non-incorporated medicines described here corroborates with previous investigations reporting that judicial decisions are not based on the current regulation of PS in the public health system (Lopes et al., 2014, Lopes et al., 2019), and most of the lawsuits could be avoided if the essential medicines lists were followed (Catanheide et al., 2016). In addition, our study revealed that medicines claimed in lawsuits were not considered essentials according to the WHO MLEM. It is reported that the main litigated products had not been incorporated in SUS because of lack of efficacy or cost-effectiveness (Chieffi et al., 2017). For universal health coverage to be possible, public policies must be adequately implemented. Litigation may be justified in some cases but should not be a rule as it is observed in Brazil.

The reasons for lack of adherence to medicines lists are multifactorial. It is reported that the prescription of non-incorporated medicines can be attributed to ineffective distribution of the lists, inadequate sensitization, lack of enforcement mechanisms, and influence of the pharmaceutical industry (Ofori-Asenso et al., 2016; Campos Neto et al., 2017). In regard to RENAME, a previous study highlighted that efforts for dissemination of the list were never a priority in Brazil; consequently, noncompliance is not an unexpected result that could potentially impair the organization of PS (Osorio-de-Castro et al., 2018).

Surprisingly, while strategies involving training about PS are incipient (Gerlack et al., 2017), the advertisement of medicines is unregulated in SUS. Medicines information from industry freely circulates in the public health system. Only 24.2% of the municipalities have norms to regulate visits from representatives of laboratories, and 32.8% regulates the distribution of free samples in health units (Costa et al., 2017). As a consequence, health professionals’ view about PS may be distorted. A nationwide study evaluating physicians’ awareness about essential medicines lists revealed they do not have adequate information and also perceive that the composition of the lists is not adequate for clinical demands (Magarinos-Torres et al., 2014).

In the present study, the implementation of educational visits was well accepted by health professionals, and it was even suggested that it should be expanded to include medicines provided by specialized component, reinforcing the importance of improving access to information about medicines provided in SUS.

In regard to users, the World Health Organization highlights the importance of civil society participation in planning and evaluation of health system services (De Savigny and Adam, 2009). In addition, based on a survey with actors from Latin America and Caribe, strategies that empower the society about the right to health were considered very important to approach medicines litigation (Pinzón-Flórez et al., 2016). For this purpose, educational strategies involving users of the public health system are needed to promote engagement and improve access to PS in SUS. This is especially relevant considering that in judiciary, health professional, and patient organizations’ perceptions, medicine selection and clinical guidelines do not meet population needs (Magarinos-Torres et al., 2014; Vargas-Pelaez et al., 2019).

The activities developed on this study revealed interesting aspects related to users’ views about medicines availability. Most participants tended to believe that the medical prescriptions should define the supply of medicines in the public health system, and a higher number of products would improve care in SUS. In contrast, there were also complaints about drug shortages, which may sound contradictory since increasing the number of medicines in REMUME will potentially make management of supply chain even more challenging.

This study is a partnership university–community which developed and implemented an innovative approach to address medicines litigation. Considering the context, how to plan and develop strategies to mitigate the threats imposed by lawsuits? The present characterization of litigation is similar to the previous studies conducted in Brazil (Oliveira et al., 2020), while others diverge, for example, by demanding mainly incorporated medicines (Freitas et al., 2020). The lack of a national database of lawsuits hinders a comprehensive overview of the phenomenon and, consequently, the generalization of the results. Nevertheless, the strategies described can guide future policies to approach litigation in different regions of the country.

One plausible explanation presented here is that prescribers demanded medicines outside the funded lists because they are not provided with adequate information about what is offered in the public system. Therefore, educational strategies are key to improve access to medicines. In spite of that, the use of educational visits for dissemination of medicine lists and strategies promoting dialogue with health system users about PS have not been reported previously.

Despite the acceptability of the strategies, equally from health workers’ and users’ perspectives, some barriers were identified. In relation to prescribers, it was challenging to schedule educational visits with the health unity manager and adjusting with the number of consultations of each physician. In contrast, with group conversations, the lack of regular educational activities and an adequate place for discussions were the main difficulties faced.

The present study has some limitations. The lack of an electronic database with information related to medicines litigation imposed challenges to data collection. Additionally, most of the lawsuits did not describe properly the reasons for litigation. Thus, it was not possible to identify, for most of the lawsuits, if the litigant had already used listed medicines before demanding non-incorporated products. Furthermore, the strategies did not include actions addressed to the judiciary sector; thus, they are not comprehensive in terms of all actors involved in litigation.

In regard to the central problem, additional influencing factors were raised, but they were not tackled yet. Medicines lists should be submitted to a careful revision to confirm if they meet the needs of the population. Likewise, failures in PS should be assessed in future investigations. The impact of the educational visits on prescribing practices is under evaluation, and the results will be reported in a future study.

It is hoped that this study can open space for new types of actions and discussion for facing medicines litigation. It is not only necessary to understand the profile of lawsuits, but it is also essential to create actions to deal with them. The group understands that empowering the society to enable them to be part of the solution to the infinite needs in the health system is a fundamental part of this process.

The effectiveness of educational interventions with different actors involved in litigation is still an issue to be investigated to support managers to implement actions to deal with this phenomenon. In addition, future studies investigating the cost-effectiveness of educational interventions would advance the comprehension of how these strategies could lead to more rational use of financial resources.

## Data Availability

The original contributions presented in the study are included in the article/Supplementary Material, further inquiries can be directed to the corresponding author.
