# Supplementary material for: Strategies to Approach Medicines Litigation: An Action Research Study in Brazil
Source: Front Pharmacol. 2021 Apr 22;12:612426. doi: 10.3389/fphar.2021.612426 (PMC8100662; doi:10.3389/fphar.2021.612426)
Supplement: Supplementary file 1 [file datasheet1.pdf]

Appendix 1

A. Group conversation with health system users - script

B. Educational outreach visits - evaluation form

C. Table 1S. Characterization of medicines lawsuits in regard to age group, medical report, reasons for litigation, legal representation and type of service that provided the prescription.

D. Table 2S. Characterization of lawsuits according to the most frequent ICD-10 blocks and medical specialties (described in at least three lawsuits).

E. Table 3S. Characterization of medicines required in the lawsuits according to the first level of ATC classification system (anatomical main group) and main dosage forms demanded

F. Table 4S. Therapeutic alternatives for non-incorporated medicines required in the lawsuits according to the National List of Essential Medicines (RENAME, in Portuguese).

A. Script - Group conversation with health system users

Question	Aim
1. The Unified Health System is obligated to ensure access to treatment for the diseases affecting the population. In your opinion, what disorders must be treated by SUS?	*Discuss the conditions that must be treated in the public health system.*
2. Do you think that the government should buy all the medicines available to treat diseases affecting their citizens?	*Discuss about the meaning of integral pharmaceutical services in the public health system and its importance for the system's sustainability.*
3. How to ensure that everyone that needs medicines will have access to them?	*Discuss the importance of medicines selection for universal access to medicines.*
4. What are the requirements for a medicine to be considered essential?	*Discuss the criteria for elaboration of essential medicines lists.*
5. Who is responsible for supplying treatments in the municipality? Who selects the medicines? Who finances the acquisition?	*Discuss the organization of pharmaceutical services and the responsibilities of federal, state and municipal governments.*
6. Present the Municipal List of Essential Medicines, inform about the Project and its purposes.	*Inform participants about the existence of medicines lists funded by the public health system and inform about the strategy to disseminate the municipal list for prescribers.*
7. In your opinion, what are the challenges to have public pharmacies at each health unit?	*Discuss the infrastructure requirements for pharmacies needed to provide an adequate environment for medicines conservation, under technical supervision of a pharmacist.*
8. Where are public pharmacies located?	*Inform about the locations of pharmacies.*
9. What documents are required to get medicines in the public pharmacies?	*Inform about the documents required for medicines dispensation in the municipality*
10. What can you do to avoid failing to get your medicines?	*Encourage participants to check medical prescription at the end of the consultation.*
11. At the pharmacy, how can I be sure that I am taking the medicine prescribed for me?	*Encourage participants to check dispensed medicines in order to prevent medication errors.*
12. If there is shortage at the public pharmacy, what options do I have to get my medicine?	*Inform about the "Programa Farmácia Popular" created to complement access to medicines through provision of free or co-financing items in private pharmacies affiliated to the program. In addition, it is also possible to encourage users to use generic medicines.*

B. Educational outreach visits - evaluation form

Health unit: ____ Date: ___/___/____						
In a scale from 1 to 5, in which **1 is totally dissatisfied** and **5 is totally satisfied**, answer the following questions:	**Totally dissatisfied**	Dissatisfied	Neutral	Satisfied	**Totally satisfied**	Uninformed
	**1**	**2**	**3**	**4**	**5**	**6**
**Questions**						
Did the facilitator succeed to present the project plainly?						
Was the duration of presentation suitable?						
Was the printed material appropriate?						
Did the facilitator answer the doubts properly?						
Did the facilitator keep an appropriate posture during the presentation?						
Was the verbal expression adequate?						

**In general:**	
Was there anything that you did not like during the visit?	**( ) Yes ( ) No** If you answered yes, could you inform what?<br>______________________<br>______________________<br>______________________<br>______________________
Could this visit be improved?	**( ) Yes ( ) No** If you answered yes, could you inform how?<br>______________________<br>______________________<br>______________________<br>______________________
Do you have any additional comments?	______________________<br>______________________<br>______________________<br>______________________

C.

Table 1S: Characterization of medicines lawsuits in regard to age group, medical report, reasons for litigation, legal representation and type of service that provided the prescription.

**Variables**	**Frequency n (%)**
**Age group**	
0 – 17 years	7 (13.7)
18 – 60 years	31 (60.8)
Above 61 years	13 (25.5)
**Medical report**	
Yes	45 (54.9)
No	37 (45.1)
**Reasons for litigation**	
Financial issues	30 (36.6)
Lack of response to the treatments available	6 (7.3)
Allergy	1 (1.2)
**Legal representation**	
Public defender	63 (81.8)
Private lawyers	14 (18.2)
**Type of service that provided the prescription**	
Public health unit	68 (82.9)
Private health unit	9 (11.0)
Public and private health units	5 (6.1)

D.

Table 2S: Characterization of lawsuits according to the most frequent ICD-10 blocks and medical specialties (described in at least three lawsuits).

**Variable**	**Frequency n (%)**
**ICD blocks**	
**Chapter II - Neoplasms**	**7 (8.5)**
CID C00-97 Malignant neoplasms of independent (primary) multiple sites	7 (8.5)
**Chapter IV - Endocrine, nutritional and metabolic diseases**	**25 (30.5)**
CID E10-14 Diabetes mellitus	21 (25.6)
CID E20-35 Disorders of other endocrine glands	4 (4.9)
**Chapter V - Mental and behavioural disorders**	**10 (12.2)**
CID F20-29 Schizophrenia, schizotypal and delusional disorders	3 (3.7)
CID F30-39 Mood [affective] disorders	4 (4.9)
**Chapter VI - Diseases of the nervous system**	**8 (9.8)**
CID G40-47 Episodic and paroxysmal disorders	5 (6.1)
**Chapter X - Diseases of the respiratory system**	**6 (7.3)**
CID J40-47 Chronic lower respiratory diseases	6 (7.3)
**Chapter XI - Diseases of the digestive system**	4 (4.9)
CID K50-52 Noninfective enteritis and colitis	3 (3.7)
**Chapter XIV - Diseases of the genitourinary system**	**8 (9.8)**
CID N17-19 Renal failure	4 (4.9)
**Medical specialties**	
Endocrinologist	23 (28.0)
Neurologist	17 (20.7)
Physician (specialty not described)	10 (12.2)
Psychiatrist	8 (9.8)
Nephrologist	6 (7.3)
Geriatrician	5 (6.1)
Pulmonologist	5 (6.1)
Oncologist	3 (3.7)
Cardiologist	3 (3.7)
Rheumatologist	3 (3.7)

E.

Table 3S: Characterization of medicines required in the lawsuits according to the first level of ATC classification system (anatomical main group) and main preparations demanded (present in at least three lawsuits). Values correspond to the percentage in relation to the total number of preparations (n=150).

Anatomical main group (ATC) and dosage forms most frequently required	Frequency n(%)
**Antiinfectives for systemic use**	1 (0.7)
**Antineoplastic and immunodulatig agents**	11 (7.3)
**Dermatologicals**	9 (6.0)
**Sensory organs**	4 (2.7)
**Systemic hormonal preparations, excl. sexual hormones and insulins**	6 (4.0)
**Antiparasitic products, inseticides and repellents**	1 (0.7)
**Blood and blood forming organs**	6 (4.0)
**Cardiovascular system**	5 (3.3)
Diosmin + hesperidin (450 mg + 50 mg)[a]	3 (2.0)
**Genito urinary system and sex hormones**	5 (3.3)
Oxybutynin chloride 5 mg	3 (2.0)
**Musculo-skeletal system**	7 (4.7)
**Nervous system**	45 (30.0)
Carbamazepine (200 mg [a, c], 400 mg [c])	3 (2.0)
Clobazam 10 mg [c]	4 (2.7)
Clonazepam (2 mg [a], 2,5 mg/mL[a, c])	6 (4.0)
Duloxetine chloride 30 mg	3 (2.0)
Pregabalin (75 mg, 150 mg)	5 (3.3)
Topiramate (50 mg, 100 mg) [b,c]	5 (3.3)
Sodium valproate (500 mg, 250 mg/5mL) [a,c]	3 (2.0)
**Respiratory System**	12 (8.0)
Formoterol fumarate + Budesonide<br>(6 mcg + 100 mcg, 12 mcg + 400 mcg [b,c])	6 (4.0)
**Alimentary tract and metabolism**	38 (25.3)
Ascorbic acid (300 mg, 1 g)	3 (2.0)
**Insulin aspart 100 UI/mL**	**9 (6.0)**
**Insulin glargine 100 UI/mL**	**14 (9.3)**
Insulin glulisine 100 UI/mL	3 (2.0)
**Insulin lispro 100 UI/mL**	**9 (6.0)**
Omeprazole 20 mg [a,c]	3 (2.0)
Total	**150 (100,00)**

[a]: Listed on REMUME; [b]: Listed on medicines provided by the state government; [c]: Listed on RENAME.

F.

Table 4. Therapeutic alternatives for non-incorporated medicines required in the lawsuits according to the National List of Essential Medicines (RENAME, in portuguese).

Dosage forms litigated	ATC	Frequency n (%)	Therapeutic alternatives	ATC	Component
**Blood glucose lowering drugs, excl. insulins**					
Dapagliflozin 10 mg	A10BK01	1 (0.4)	Metformin chloride	A10BA02	Basic
Empagliflozin 25 mg	A10BK03	1 (0.4)			
Glimepiride 4 mg	A10BB12	1 (0.4)	Glibenclamide	A10BB01	
Liraglutide 1,2 mg/mL	A10BJ02	1 (0.4)			
Liraglutide 6 mg		1 (0.4)	Gliclazide	A10BB09	
Sitagliptin phosphate 100 mg	A10BH01	2 (0.9)			
Vildagliptin 50 mg	A10BH02	1 (0.4)			
**Insulins and analogues**					
Insulin aspart 100 UI/mL	A10AD05	9 (4.0)	Insulin human	A10AB01	Basic
Insulin glulisine 100 UI/mL	A10AB06	3 (1.3)			
Insulin lispro 100 UI/mL	A10AB04	9 (4.0)			
Insulin glargine 100 UI/mL	A10AE04	14 (6.2)	Insulin human NPH	A10AC01	
Insulin degludec 100 UI/mL	A10AE06	1 (0.4)			
Insulin detemir 100 UI/mL	A10AE05	2 (0.9)			
**Antipsychotics**					
Aripiprazole 10 mg	N05AX12	1 (0.4)	Lithium carbonate	N05AN01	Basic
			Chlorpromazine chloride	N05AA01	
Aripiprazole 15 mg		1 (0.4)	Haloperidol decanoate	N05AD01	
			Haloperidol		
Levomepromazine maleate 25 mg	N05AA02	1 (0.4)	Clozapine	N05AH02	Specialized
Levomepromazine maleate 100 mg		1 (0.4)	Ziprasidone chloride	N05AE04	
			Quetiapine hemifumarate	N05AH04	
Paliperidone palmitate 75 mg	N05AX13	1 (0.4)	Olanzapine	N05AH03	
			Risperidone	N05AX08	

**Antiepileptics**					
Divalproex sodium 500 mg	N03AG01	2 (0.9)	Valproic acid	N03AG01	
			Carbamazepine	N03AF01	
			Clonazepam	N03AE01	Basic
			Phenytoin	N03AB02	
Oxcarbazepine chloride 600 mg	N03AF02	2 (0.8)	Phenobarbital	N03AA02	
			Ethosuximide*	N03AD01	
			Gabapentin	N03AX12	
Pregabalin 75 mg	N03AX16	3 (1.3)	Lamotrigine	N03AX09	
			Primidone*	N03AA03	Specialized
Pregabalin 150 mg		2 (0.9)	Topiramate	N03AX11	
			Vigabatrin	N03AG04	

**Medicines listed on RENAME, however they were not incorporated by the state government and therefore, they were not available at the specialized pharmacy in the municipality studied.*
